# gcProfileMakeR: An R Package for Automatic Classification of Constitutive and Non-Constitutive Metabolites

**DOI:** 10.3390/metabo11040211

**Published:** 2021-03-31

**Authors:** Fernando Perez-Sanz, Victoria Ruiz-Hernández, Marta I. Terry, Sara Arce-Gallego, Julia Weiss, Pedro J. Navarro, Marcos Egea-Cortines

**Affiliations:** 1 Instituto Murciano de Investigaciones Biomédicas El Palmar, 30120 Murcia, Spain; fernando.perez8@um.es; 2 Department of Biosciences, University Salzburg, 5020 Salzburg, Austria; victoria.ruiz@sbg.ac.at; 3 Genética Molecular, Instituto de Biotecnología Vegetal, Edificio I+D+I, Plaza del Hospital s/n, Universidad Politécnica de Cartagena, 30202 Cartagena, Spain; marta.terry@edu.upct.es (M.I.T.); julia.weiss@upct.es (J.W.); 4 Vall d’Hebron Institute of Oncology, 08035 Barcelona, Spain; sara.arcegallego@gmail.com; 5 DSIE Cuartel de Antiguones, Plaza del Hospital s/n, Universidad Politécnica de Cartagena, 30202 Cartagena, Spain; pedroj.navarro@upct.es

**Keywords:** automatic classification, gcProfileMakeR, constitutive metabolome, non-constitutive metabolome, machine learning, floral organ identity, R package, circadian clock

## Abstract

Metabolomes comprise constitutive and non-constitutive metabolites produced due to physiological, genetic or environmental effects. However, finding constitutive metabolites and non-constitutive metabolites in large datasets is technically challenging. We developed gcProfileMakeR, an R package using standard Excel output files from an Agilent Chemstation GC-MS for automatic data analysis using CAS numbers. gcProfileMakeR has two filters for data preprocessing removing contaminants and low-quality peaks. The first function NormalizeWithinFiles, samples assigning retention times to CAS. The second function NormalizeBetweenFiles, reaches a consensus between files where compounds in close retention times are grouped together. The third function getGroups, establishes what is considered as Constitutive Profile, Non-constitutive by Frequency i.e., not present in all samples and Non-constitutive by Quality. Results can be plotted with the plotGroup function. We used it to analyse floral scent emissions in four snapdragon genotypes. These included a wild type, *Deficiens nicotianoides* and *compacta* affecting floral identity and *RNAi:AmLHY* targeting a circadian clock gene. We identified differences in scent constitutive and non-constitutive profiles as well as in timing of emission. gcProfileMakeR is a very useful tool to define constitutive and non-constitutive scent profiles. It also allows to analyse genotypes and circadian datasets to identify differing metabolites.

## 1. Introduction

Plants, fungi, bacteria and animals emit complex mixtures of Volatile Organic Compounds (VOCs) forming blends or scent profiles. Scent profiles are considered as the core volatile metabolome of a tissue, organ or organism. Floral scent is a combination of VOCs that is emitted by flowers in order to attract pollinators and deter pests [1].

Plants emit volatiles from different organs such as roots, leaves and flowers [2]. Different plant organs emit different combinations of volatiles, giving rise to the so called green or leaf scent and floral scent [3]. The production of floral VOCs is coordinated by two layers. Floral organ development occurs by activation of a set of MADS-box genes in a combinatorial fashion called floral organ identity genes. Proper petal formation occurs by activation of the so-called B function genes, such as *DEFICIENS* and *GLOBOSA* or *APETALA3* and *PISTILLATA* in Arabidopsis [4]. Down-regulation of *DEF* in snapdragon by RNAi causes a decrease in volatile synthesis indicating that floral scent is downstream of the organ identity genes [5]. A second layer of control occurs via the circadian clock. Floral volatiles are typically emitted in a circadian fashion. Many plants produce scent preferentially during the day such as snapdragon or rose while other emit preferentially during the night such as petunia [6,7]. Indeed, down regulation of clock genes such as *CHANEL* and *GIGANTEA1* in petunia or *LATE ELONGATED HYPOCOTYL* in snapdragon cause important changes in emission timing [8,9,10].

Volatile emissions play important biological functions. The study of floral scent has shown that as VOCs emitted by a given species or organ is generally robust, they can be used for phylogenetic reconstruction [11,12,13]. This suggests that for a given species a standard or constitutive scent profile can be consistently identified. In contrast, induced volatile emissions are associated to processes such as abiotic stress or biotic interactions [14,15,16]. These VOCs that may not be found robustly on a given scent profile, become constitutive under these new circumstances. Well known examples of non-constitutive metabolites that become constitutive include salicylic acid in response to pathogens [17,18], jasmonic acid to light cues [19] or indole in maize in response to herbivores [20,21]. Another type of variability is purely genetic, i.e., differences in presence/absence of one or several VOCs between mutants, accessions, varieties or species [11,13,22]. As plant VOC chemodiversity is very high [23], identifying differential VOCs becomes really challenging. A third important factor affecting VOC composition and quantities is temperature. Indeed hot and cold temperatures have a major effect on VOCs emitted by plants [24,25,26]. Thus, having a clear picture of the composition of VOCs that can be considered as constitutive profile becomes experimentally challenging even for very controlled experiments. In this context, the bioinformatic aid towards clear datasets comprising constitutive and non-constitutive compounds is of great help.

Automatic gas chromatography mass spectrometry (GC-MS) data annotation is relatively easy nowadays thanks to the development of mass spectra libraries and programs created for this purpose [27]. The structure of a metabolome i.e., the specific metabolites that comprise a sample, and their quantities are two aspects defining metabolomics [28]. However, reaching a consensus among samples of which compounds are comprising the constitutive metabolome and which form the non-constitutive metabolome is mainly performed manually, and criteria are not always obvious. An additional issue is the complexity of names given to a single chemical compound. In many cases, they include a common name, a chemical structure and sometimes isomers. The Chemical Abstract Service Number or CAS number is a single identifier that allows unambiguous assignation of a chemical structure. Thus the adoption of CAS-number defined metabolomes is the most appropriate way to handle metabolomics raw data in a suitable format for FAIR data management where data can be reanalysed [29].

There are bioinformatic platforms where LC/MS data can be completely analysed such as Metaboanalyst [30,31]. However, the current trend is towards open-source freely available software [32]. The bioinformatic handling of metabolomic data has several discrete steps. Peak detection starts with baseline correction stage of peak detection. Deconvolution is performed in order to merge overlapping peaks, small shoulders on peaks and fragments originating from the same metabolite. This step is sometimes performed directly by the software of the LC/MS or GC/MS machine. Some software packages have been developed for automatic deconvolution such as eRaH [33]. Finally, retention times of different samples are aligned to peaks. As retention times may vary between samples, different methods of alignment have been devised [27]. The complete process is called workflow [32]. Some web software packages such as Metflow perform this type of data pretreatment [34]. There are a number of software packages and platforms that allow extensive comparisons of a sample to datasets of retention times such as METLIN [35]. However, free software packages whereby large sample datasets can be compared with each other to identify common and differing metabolites have not been published. Indeed the problem has been addressed previously by a semiautomated strategy based on hierarchical curve resolution for LC/MS data [36], but a software package to perform this procedure is not available.

Here we provide an R-package that uses as inputs Excel spreadsheet files produced by GC-MS apparatus such as Agilent Chemstation. gcProfileMakeR determines the core metabolome and non-constitutive compounds emitted by a group of samples. It uses CAS numbers produced by GC-MS apparatus to sort the lists of common i.e., constitutive metabolites, and those appearing only on part of the data set i.e., non-constitutive metabolites or divergent between samples or treatments.

## 2. Results

The full implementation of non-targeted metabolomics produce very large lists of liquid and/or gas chromatograms comprising hundreds of compounds [37]. Oftentimes, the number of compounds described undergo an arbitrary cut-off as major and minor components, based on percentages of detected emission, thus focusing further analysis on a subset of the metabolome. Another reason to focus on a subset of metabolites is that comparison between samples is performed manually. Thus, comparing a set of twenty to thirty samples may take months. We developed gcProfileMakeR, a tool accelerating the actual identification of common compounds in a set of samples. It uses reproducible criteria for downstream processing and data reusability. gcProfileMakeR was developed as an R package as R is open source, and the scientific community, especially biology, is doing a massive use of it. gcProfileMakeR determines the core metabolome and non-constitutive compounds present in a set of samples, thus allowing extensive data exploration. This library has been used to analyse several biological datasets, including the characterization of post-harvest conditions on scent emission in narcissus cut flowers [38].

### 2.1. gcProfileMakeR Input Data

Some packages are recommended to be pre-installed in R before gcProfileMakeR runs: readxl, plyr, stringr, dplyr, tidyr, ggplot2 and egg.

**Figure 1:**
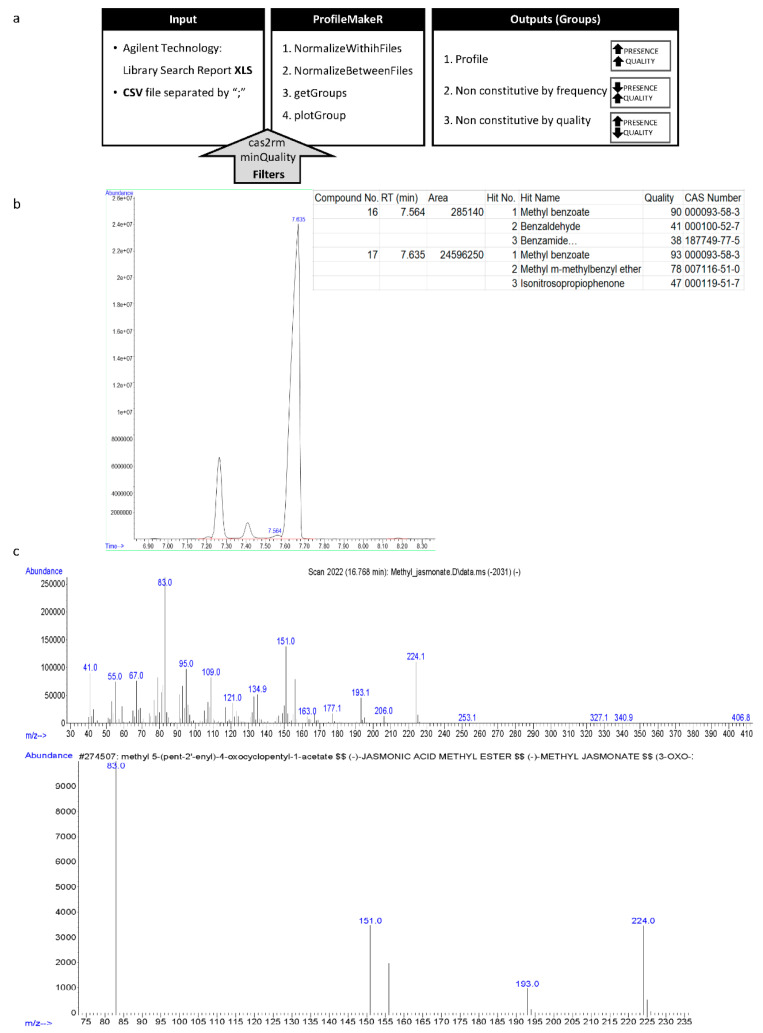
(**a**) gcProfileMakeR pipeline. This library accepts Excel (.xls) and. csv files as input data. The first function, NormalizeWithinFiles, reads the data and groups compounds with similar retention time (RT) and common CAS numbers. Users also can apply two filters: cas2rm (compound/s to exclude) and minQuality (minimum quality). NormalizeBetweenFiles groups compounds with similar RT in all files, with the most representative CAS number. getGroups determines the constitutive and non-constitutive profiles (i.e., metabolic profile) by frequency and quality, which are chosen by the user. Finally, plotGroup creates a graphic the constitutive, non-constitutive by frequency and/or non-constitutive by quality. (**b**) A standard chromatogram where two close peaks are integrated separately by default and dataset corresponding to peaks, where the identity with highest probability of the peaks is the same, methyl benzoate (CAS number 93-58-3). (**c**) Mass spectra of methyl jasmonate (CAS No: 39924-52-2), a commercial standard (upper panel) and mass spectral database (lower panel) Willey10th-NIST11b.

gcProfileMakeR uses two types of raw data: either XLS data files obtained directly from Agilent Chemstation software (Library Search Report) or CSV files (Figure 1a). An example dataset can be retrieved within the library. The gcProfileMakeR can be downloaded from https://github.com/fpsanz/gcProfileMakeR (accessed on 30 March 2021).

GC basic data contains information for each integrated peak about retention time (RT) and area of the peak. Mass spectra alignment with available libraries (MS libraries) allows to identify the compounds present in the sample with a certain degree of confidence (quality). Annotated compounds (hits) are listed according to the quality of the match between the mass spectra obtained and the mass spectra listed in the MS library. Hits are specified by chemical names of compounds and the CAS Registry Number associated to the hit/compound. CAS numbers are specific for a compound whereas chemical names are redundant and may imply different isomers or molecules. gcProfileMakeR works with RT, qualities and CAS numbers in order to provide lists of compounds identified by CAS numbers, areas and qualities. Chemical names are linked to the CAS numbers as they are understandable by scientists.

### 2.2. gcProfileMakeR Data Pretreatment Filters

Two filters can be applied to pretreat data (Figure 1a).

- **cas2rm**. The first one, cas2rm, will sort out any CAS number defined by the user, thus allowing the elimination of known contaminants, or compounds that are ubiquitous and complicate further analysis.
- **minQuality**. The second filter, minQuality, eliminates hits, either first or secondary, with a mean quality below a defined level. Specific retention peaks may be filtered out from the profile if being too strict (e.g., = 95). It allows to use a strategy of low strictness at the integration step and explore the data, decreasing the threshold to define a complete metabolome.

### 2.3. gcProfileMakeR Data Pretreatment Filters

gcProfileMakeR uses four functions (Figure 1a).

**Figure 2:**
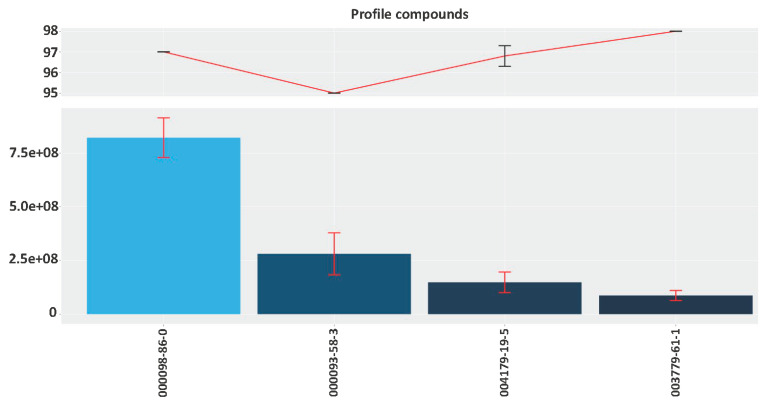
plotGroup function. This graph shows the constitutive profile by frequency of the wild-type snapdragon at ZT9 (Zeitgeber time). The x-axis shows the CAS number of volatile organic compounds. The upper part displays the average quality of volatiles (red line) and the lower part of the graph indicates the average areas of compounds (blue bars), that are plotted in decreasing order. Whiskers show the standard deviation of quality (upper part) and area (lower part).

- **NormalizeWithinFiles.** The first function NormalizeWithinFiles, analyses each file/sample assigning for each retention time a set of possible hits (compounds). Peak areas of the same compounds with an identical CAS number found in different RTs, will be added (Figure 1b).
- **NormalizeBetweenFiles**. The second function NormalizeBetweenFiles, reaches a consensus between files in such a way that the same compounds separated in relatively close retention times are grouped together. This is important as even a standard does not always run at the precise same retention time. Thus peaks that appear very close in retention and have the same CAS number are grouped together.
- **getGroups**. The third function getGroups, establishes what is considered as “Profile”, “Non-constitutive by Frequency” and “Non-constitutive by Quality”. The Profile refers to those compounds that are present in all samples and can be considered constitutive. Non-constitutive by Frequency is a list of compounds present in several, but not all samples of a given class i.e., a species, a mutant or a treatment. The rationale behind including a Non-constitutive by Quality list is that some compounds, even as chemical standards, give low quality due to poor representation in MS libraries, for instance methyl jasmonate (Figure 1c). Indeed a compound may be present in all samples but with low quality. Frequency and quality default thresholds can be adjusted, thus allowing data exploration.
- **plotGroup**. Results can be plotted with the function plotGroup (Figure 2). In this function, compoundType parameter can be adjusted in order to get profiles (p), non-constitutive by frequency (ncf) or non-constitutive by quality (ncq). Results are plotted according to the average area and quality of each compound grouped in each category. The graphic obtained is in HTML format and allows, by pointing at the columns, to see the actual compound names that are linked to a CAS number (Figure 2). Pointing at the quality percentages it shows the error rates of the quality for a given CAS number. This facilitates working with the graphics. They can also be saved as png.

Default values have been tested with different sets of samples and number of samples and have proved the best outputs when compared to manual annotation (data not shown). The output of gcProfileMakeR are three mutually exclusive lists of compounds. The first set of compounds listed as “Profile” are those compounds which appear in all the samples of a given type i.e., genotype and/or treatment and which have a high matching quality: above a percentage of samples defined by the researcher. Compounds listed as “Non-constitutive by Frequency” are metabolites with a high mean-quality score (default: >85%) in the MS analysis but present in less than the percentage of the samples defined previously by the researcher (Figure 1a). Finally, compounds listed as “Non-constitutive by Quality” are metabolites with a low mean-quality (default: <85%) that are in at least 30% of the samples (default value) (Figure S1). All frequency and quality thresholds can be adjusted for stringency thus allowing data exploration.

### 2.4. Testing gcProfileMakeR in Floral Organ Identity Mutants and RNAi:AmLHY

We have experimentally validated gcProfileMakeR using a set of *Antirrhinum majus* mutants, transgenic and wild type plants.

We analyzed four datasets of floral volatiles, 16 samples corresponding to Sippe 50 wild types, nine produced by the mutant *def^nic^*, 35 corresponding to the mutant co and 40 corresponding to several independent *RNAi:AmLHY* lines. We used a list of possible contaminants, which might proceed from the twister absorption matrix (Table S1), and cas2rm to eliminate from our results any CAS numbers corresponding to siloxane or related derivatives. The rationale behind gcProfileMakeR is to obtain a set of metabolites, in this case scent VOCs, that are common to a given set of samples and we call profile, or constitutive metabolome. But we also want to have a second list of VOCs that are not found in all samples and nevertheless are produced. This are the non-constitutive by quantity.

**Figure 3:**
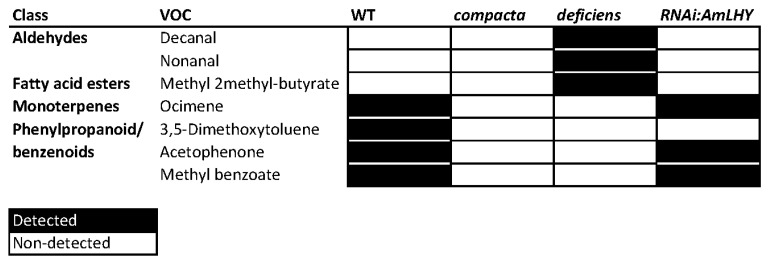
Heat map of constitutive by frequency scent profile of wild-type snapdragon (Sippe 50), the mutants *co*, *def^nic^* and the transgenic lines RNAi:AmLHY. We set minQuality to 80% (NormalizeWithinFiles function). Constitutive profile comprises those compounds present in 100% of analyzed samples. Volatile compounds are clustered by class. Black and white colors denote a detected and a non-detected compound, respectively.

**Figure 4:**
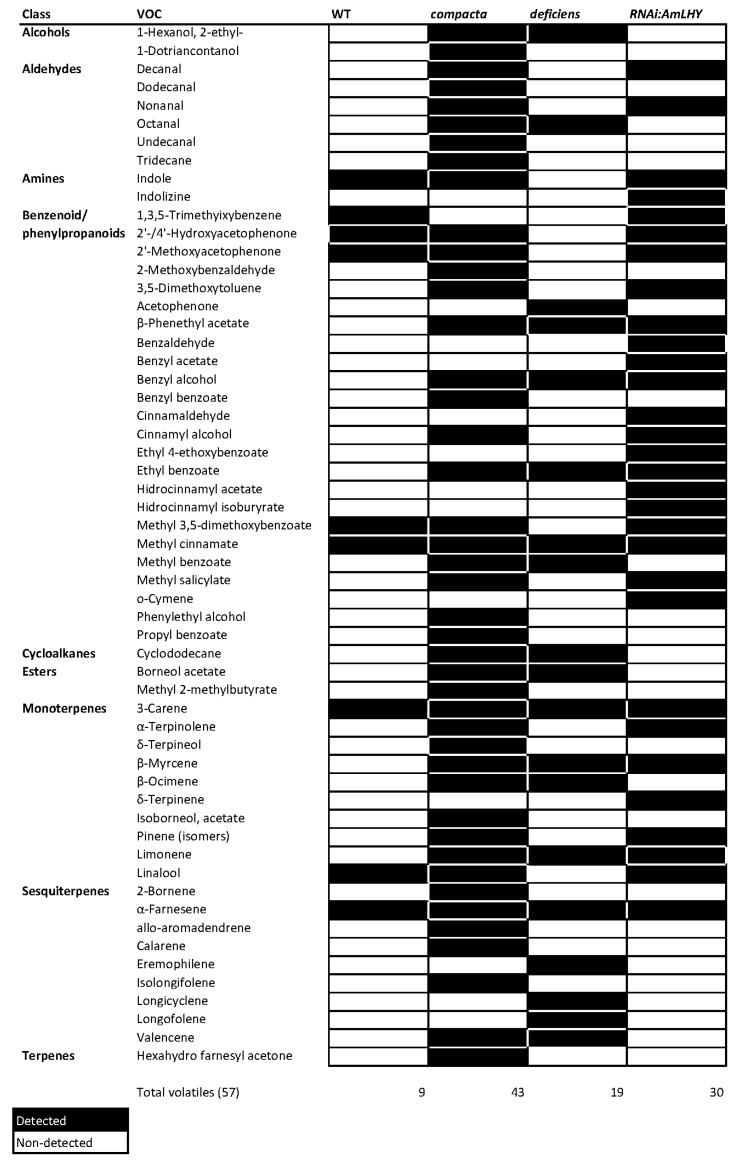
Heat map of non-constitutive by frequency scent profiles of wild-type snapdragon (Sippe 50, WT), the mutants *compacta* and *deficiens-nicotianoides* and the transgenic line *RNAi:AmLHY*. We set minQuality to 80% (NormalizeWithinFiles function). Non-constitutive profile comprises those compounds that present in 99% or less of analyzed samples. Volatile compounds are clustered by class. Black and white colors indicate a detected and a non-detected compound, respectively. Total volatiles, indicates the number of detected volatiles among snapdragon genotypes.

Using gcProfileMakeR we obtained a comprehensive profile present in all samples of a given genotype (pFreqCutoff = 1.0) setting minimum quality to 80% (minQuality = 80). This strict cutoff gave a constitutive profile in wild type plants comprising four volatiles: the monoterpene ocimene, and the phenylpropanoids 3,5-dimethoxytoluene, acetophenone and methyl benzoate (Figure 3). There were nine additional non-constitutive compounds emitted giving in total of 13 VOCs produced by a wild type flower (Figure 4). Surprisingly, *co* mutants that have a mild floral identity phenotype, did not emit a single VOC as constitutive scent but rather emitted 44 VOCs as non-constitutive metabolites by frequency (Figure 3 and Figure 4). When we analyzed *def*-*nic* flowers, a stronger mutation than *co*, we found two aldehydes, decanal and nonanal, and the fatty acid ester methyl 2-methyl butyrate as constitutive, together with an additional set of 19 non-constitutive VOCs. Thus, the constitutive profile of *def-nic* flowers did not share a single volatile with the WT flowers. The WT non-constitutive profile shared typical floral scent components such as indole, methyl cinnamate and linalool, with *co*. However, the only non-constitutive VOCs shared by the three genotypes were 3-carene and α-farnesene. These results show that mutations in the organ identity genes *def-nic* and *co* cause a major change of the VOC emission of the flower. Organ identity genes appear to define a shortlist of VOCs comprising the floral scent.

We used three independent lines (*RNAi:AmLHY*) where the circadian clock gene *AmLHY* is silenced [8]. These transgenic lines emitted three constitutive VOCs, ocimene, acetophenone and methyl benzoate, coinciding with WT flowers (Figure 3 and Figure 4). But again, the number of VOCs emitted as non-constitutive was larger than WT counting up to 30 (Figure 3 and Figure 4). The non-constitutive VOC profile of *RNAi:AmLHY* was substantially richer than in wild type flowers including VOCs found only in these lines such as benzaldehyde, benzyl acetate and benzyl alcohol, cinnamaldehyde, o-cymene or terpinene.

In order to verify if these findings hold true, we reanalyzed the data, setting the threshold for constitutive components to 70% (Figures S2 and S3). As expected, the amount of volatiles comprising the constitutive set increased to seven in WT, five in *co*, five in *def-nic* and 14 in *RNAi:AmLHY*. This increase still showed clear differences between genotypes in terms of the actual set of volatiles emitted (See below).

The combination of constitutive and non-constitutive profiles was reflected in the complexity of the chromatograms. Indeed, chromatograms corresponding to wild type flowers appeared less complex than *compacta* mutants, or *RNAi:AmLHY* (Figure S4). Altogether, we found 57 different volatiles emitted by WT and the different mutants or RNAi lines.

**Figure 5:**
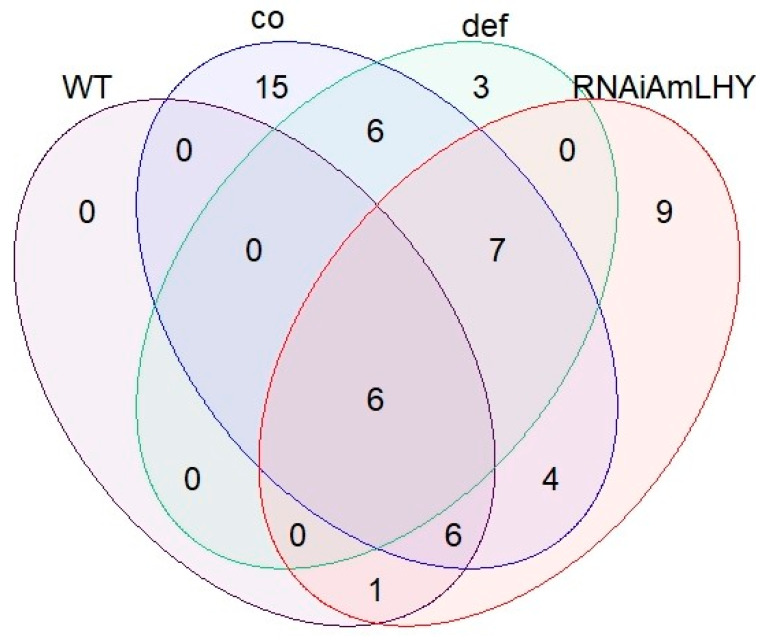
Venn diagram showing overlapping volatiles organic compounds detected in wild type (purple), *co* (blue), *def^nic^* (green) and RNAi:AmLHY (red) snapdragon flowers. These comprise 57 VOCs, i.e., the sum of profile and non-constitutive by quantity.

We plotted the gcProfileMakeR outputs for all the volatiles together (constitutive and non-constitutive) as a Venn diagram to identify shared and unique volatiles. We found that the different genotypes showed distinct compounds (Figure 5). This type of analysis may help identify the actual molecular mechanisms that coordinate specific VOC production. Six common volatiles were produced by all genotypes comprising carene, acetophenone, methyl benzoate, methyl cinnamate, farnesene and ocimene, while other volatiles were exclusively produced by one genotype.

### 2.5. Analysis of Volatile Metabolic Pathways with gcProfileMakeR Outputs

**Figure 6:**
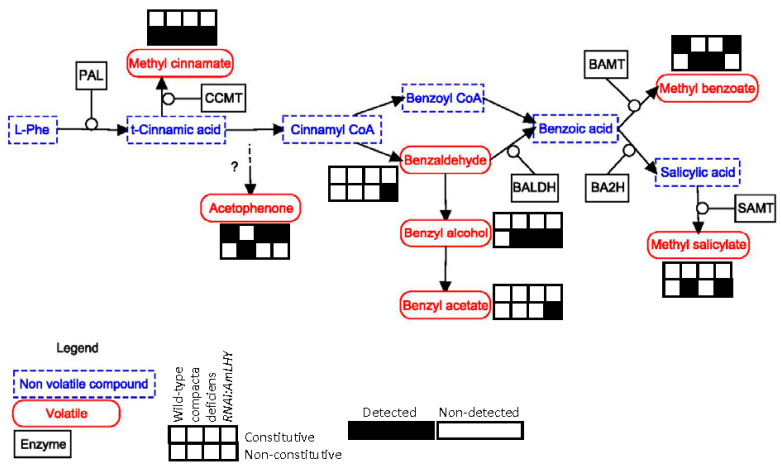
Benzenoid/phenylpropanoids schematic pathway. Detected and non-detected volatiles are shown as follow: first row refers to constitutive profiles and second row to non-constitutive by frequency profiles. Detected compounds are depicted by black and not detected by white. Each column represents a snapdragon group: wild-type (1st), co (2nd) and defnic (3rd) and transgenic lines RNAi:AmLHY (4th). PAL: phenylalanine ammonia lyase, CCMT: cinnamic acid carboxyl methyl transferase, BALDH: benzaldehyde dehydrogenase, BA2H: benzoic acid 2-hydroxylase, BAMT: benzoic acid carboxyl methyl transferase, SAMT: salicylic acid carboxyl methyl transferase.

**Figure 7:**
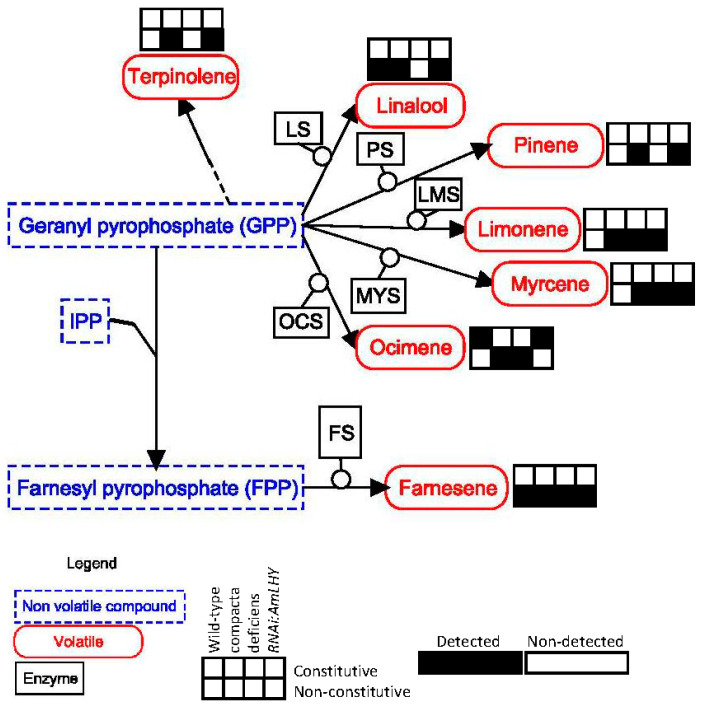
Terpenoids schematic pathway. Representations are like in Figure 7. LS: linalool synthase, PS: pinene synthase, LMS: limonene synthase, MYS: myrcene synthase, OCS: ocimene synthase, FS: farnesene synthase, IPP: isopentenyl diphosphate.

We plotted the schematic pathway of benzenoid/phenylpropanoids and terpenoids pathways (Figure 6 and Figure 7), indicating which group of volatiles is emitted by different genotypes and its frequency among the analysed population [2]. Methyl cinnamate was common to all genotypes analysed but was not a constitutive volatile (Figure 6). Benzaldehyde appeared only in *RNAi:AmLHY* lines. However, we found benzyl alcohol in *def-nic*, *co* mutants and the silenced *RNAi:AmLHY* plants but not in wild type. This indicates that benzaldehyde is partly diverted towards the synthesis of benzyl alcohol. Further down the pathway, we found benzyl acetate in *RNAi:AmLHY* indicating that the repression of this pathway may be coordinated by *AmLHY*. These results suggest a preferred route: the volatiles benzaldehyde and benzyl alcohol are not found in the constitutive profile of any snapdragon group whereas methyl benzoate is constitutively emitted in wild-type and *RNAi:AmLHY* lines but not in mutants affecting floral organ identity.

The monoterpenes terpinolene, linalool, pinene, limonene, myrcene and ocimene share the precursor geranyl pyrophosphate (Figure 7). Terpinolene, pinene, limonene and myrcene were not present in the constitutive profile of analysed plant groups whereas linalool showed a constitutive emission in WT and *RNAi:AmLHY* and ocimene, in all plants except in *def-nic*. Farnesene appeared as non-constitutive in all genotypes indicating that this pathway is not affected by the mutants analysed.

### 2.6. Analysis of Volatile Circadian Emission with gcProfileMakeR

**Figure 8:**
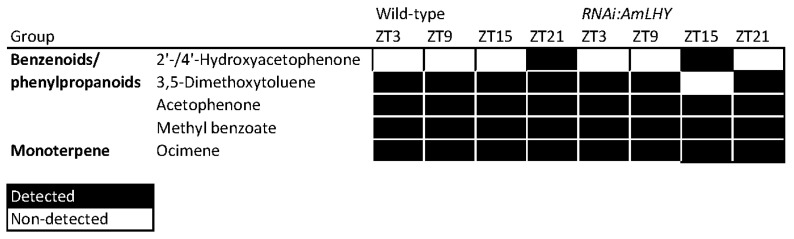
Constitutive scent profile of wild-type and transgenic RNAi:AmLHY snapdragons at four different time-points, denoted as ZT (zeitgeber time) 3, 9, 15 and 21. ZT0 represents the time of lights on and ZT12, lights off. We set minQuality to 80% (NormalizeWithinFiles function). Constitutive profile includes VOCs present in 100% of analyzed samples. Volatiles are listed according to their class. Black indicates detected compounds and white, non-detected compounds.

Most of the previous works about the function of circadian clock genes on scent emission have described the effect of down regulation of a gene on a small subset of VOCs. Here we analysed the complete volatilome at four times during the day. There is a trade off as sampling for very short periods may help determine daily changes in emission with a higher level of resolution in terms of rhythmicity [39]. However when volatiles are sampled for shorter periods they show simpler chromatograms as VOCs produced in smaller amounts may not be captured. Thus, we took four sampling times per day. When we used highly stringent parameters to establish the constitutive profile of 100% on all samples we found that WT and *RNAi:AmLHY* plants shared a set of volatiles emitted throughout the day (Figure 8). These were 3,5 dimethoxytoluene, acetophenone, methyl benzoate and ocimene. However, at ZT 21, we detected 2′-/4′-hydroxyacetophenone in WT and this same compound was found at ZT15 in the *RNAi:AmLHY*, demonstrating a function of *AmLHY* in the timing of emission. When we relaxed the stringency criteria to 70% for constitutive volatiles (Figure S4), we found that the number of volatiles emitted by *RNAi:AmLHY* was much larger than those emitted by wild type plants. The changes in rhythmic expression became apparent in all the different VOCs classes, aldehydes, amines, benzenoids, mono- and sesquiterpenes. Decanal and nonanal, absent in wild type, appeared in the late evening (ZT21) and early morning (ZT3) in *RNAi:AmLHY*. A loss of periodicity was found for methyl 3,5-dimethoxybenzoate, emitted in WT only during the light period, and constitutively by *RNAi:AmLHY*. Interestingly, linalool appeared to be emitted in WT during the very late evening (ZT21) and early morning (ZT3) becoming constitutive in *RNAi:AmLHY* i.e., it lost its periodic emission. We can conclude that by analysing the data at different levels of stringency we could find patterns of VOC emission that appeared to be coordinated by *AmLHY*.

### 2.7. Analisys of gcProfileMakeR Outputs

An important question is if the constitutive profile list of volatiles of the different genotypes can be further analysed to obtain insights about the classified compounds. We defined the “constitutive scent profile”, which contained for each snapdragon genotype the volatiles that were present in all samples. Combining the profile of every group, we obtained a list of seven VOCs: the aldehydes decanal, nonanal, the benzenoids 3,5-dimethoxytoluene, acetophenone and methyl benzoate, the ester methyl 2-methylbutyrate and the monoterpene ocimene (Figure 3). This restrictive analysis revealed four different aroma blends resulting from a combination of seven volatiles (Figure 3). We further analysed these profiles by multivariate analysis (see below). Notice that a population or group with certain non-constitutive volatile does not imply that is completely absent: gcProfileMakeR allows the researcher to choose and define a profile.

**Figure 9:**
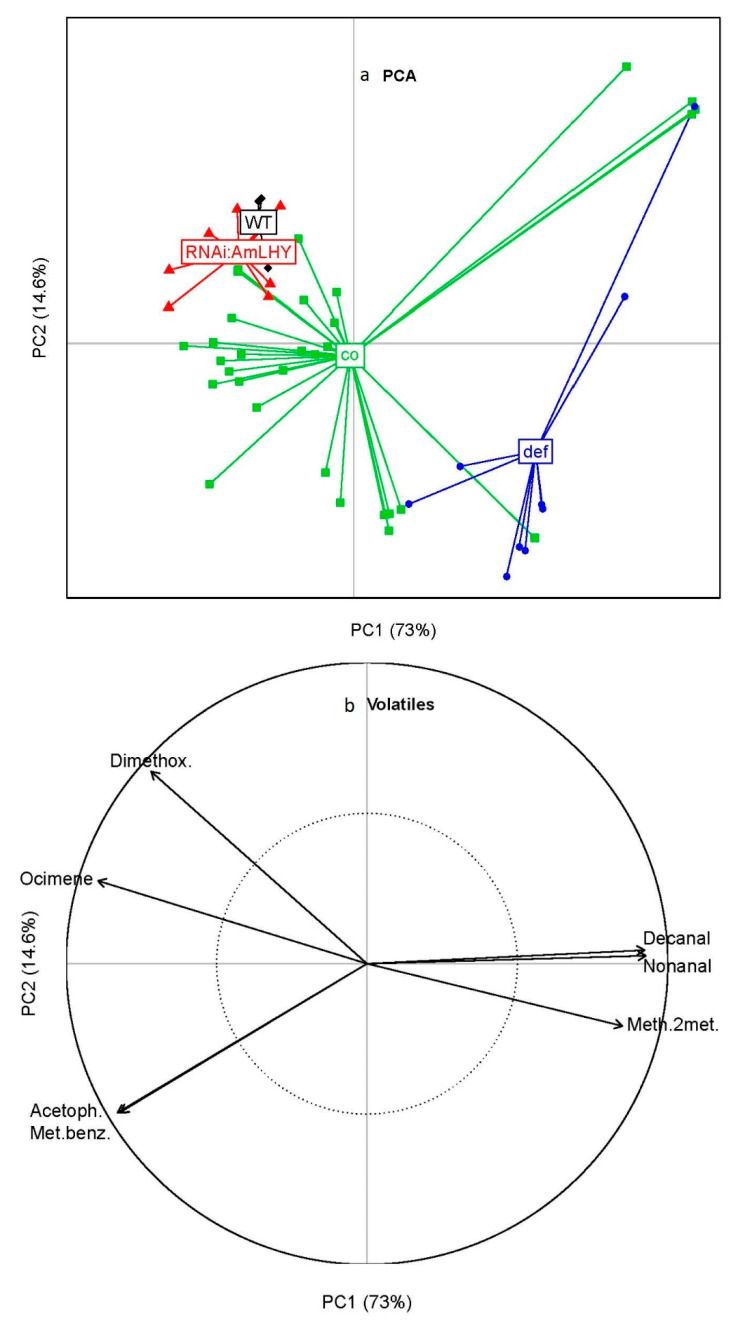
Principal component analysis of snapdragon groups based on their emitted compounds. The axis PC1 and PC2 explains the 73% and 14.6% of the total variance respectively (**a**). Correlation circle plot that represents the contribution of volatiles (**b**). PC. Principal Component, co: *compacta*, def: *deficiens*, WT: wild-type, Dimethox.: 3,5-dimethoxytoluene, Acetoph.: acetophenone, Met.Benz.: methyl benzoate, Meth.2met.: methyl 2-methylbutyrate.

We obtained the integrated peak area of seven volatiles, which were constitutively emitted by snapdragon groups (Figure 3), from the AuxTable file. These VOCs included decanal, nonanal, 3,5-dimethoxytoluene, acetophenone, methyl benzoate, methyl 2-methylbutyrate and ocimene. In order to identify snapdragon groups based on their aroma blends, we performed a principal component analysis (PCA) [40]. The principal component (PC) 1 and 2 explained 87.6 % of the variance observed in scent emission. As described previously (Figure 3) and focusing in constitutive volatiles, wild-type and *RNAi:AmLHY* aroma profiles were similar. In contrast, *compacta* and *deficiens* mutants differed from each other and from wild-type and transgenic snapdragons (Figure 9a). The correlation plot (Figure 9b) revealed that methyl benzoate and acetophenone were positively correlated and along with 3,5-dimethoxytoluene and ocimene “clustered” wild type, *RNAi:AmLHY* and *compacta*. On the other hand, nonanal and decanal were also positively correlated and with methyl-2-methylbutyrate defined *deficiens* scent profile. These results revealed that a strict cutoff, as we have defined for constitutive scent profile, could be used for classifying populations or groups.

We also performed a classification analysis using four Machine Learning algorithms that are widely used: k-Nearest Neighbours (k-NN), Naïve Bayes Classifier (NBC), Random Forest (RF) and Support Vector Machine (SVM). For SVM, we used three different transformations: linear, radial and polynomial. In addition, we compared two scent profiles defined by their frequency, compounds detected in all flowers or constitutive profile (pFreqCutoff = 1.0) and detected in 70% or more samples (pFreqCutoff = 0.7). These two profiles comprised 7 and 16 volatiles, respectively (Figure 3, Figure S2), and we will refer to them as group 1 and group 2. We used the caret R package to perform and compare the algorithms. (Table 1). The algorithm ranking was similar for both aroma scents. Based on accuracy and Kappa coefficient, RF and SVM with a polynomial kernel, were the best models whereas NBC and SVM with a radial kernel, the worst. Moreover, accuracy and kappa values were slightly higher across all algorithms in group 2 (Table 1).

Comparing the RF output, we found that the error out of bag (OOB) was 14.58% in group 1 (pFreqCutoff = 1.0) and 4.17% in group 2 (pFreqCutoff = 0.7) (Table 2). RF also provides a rank list with the accuracy in which a predictor, a volatile in our case, can be used for classification. For the group 1, the most important compounds were acetophenone, 3,5-dimethoxytoluene and methyl benzoate whereas for group 2 were nonanal, methyl-2-methyl butyrate and farnesene (Table 3).

### 2.8. Timescale Improvement Using gcProfileMakeR

The complete dataset used in this study comprises 100 GC-MS samples and a total of 3238 peaks comprising 100 samples and 57 different VOCs. This dataset had been previously curated by the cas2rm and the minQuality filters in an automatic fashion. The manual filtering for quality and removal of low quality peaks makes it close to 4000 peaks. These were mainly siloxanes and derivatives. The manual sorting of the dataset may take several months for a well-trained person. Using the built-in R function Sys.time(), the complete dataset was analyzed in 27.6 s on a Lenovo Legion Y250 laptop (Lenovo Group Limited, Registered Office: 23rd Floor, Lincoln House, Taikoo Place, 979 King’s Road, Quarry Bay, Hong Kong S.A.R. of China; purchased on Amazon.es) equiped with an Intel^®^Core™-i5-/300HQ CPU at 2.50 GHz and 16.0 GB of RAM. Using the same computer, the *co* dataset of 35 samples ran in 6.50 s. This speed of analysis allowed an extensive data exploration that is otherwise time consuming and, when performed manually, prone to errors.

## 3. Discussion

In this work we present an R package that helps classify metabolites from large datasets. Currently, the development of CRISPR/Cas9 technologies, massive analysis of natural variation and classically mutagenized populations bring the opportunity to understand the control of plant metabolism. However, a major burden is that metabolome analysis is performed mostly manually.

gcProfileMakeR is a freely available R package that allows the identification of common volatiles for a set of samples and those that differ with other samples or are not found as constitutive components.

We have coded a package that works with CAS numbers. As CAS numbers define a single compound, that may have different names such as chemical formula or common name, they avoid issues in terms of data traceability. Previous open software performing peak identification such as TargetSearch, uses ion extraction for peak finding [41] while in gcProfileMakeR, this is performed by the GC/MS software with the NIST library and CAS numbers are used. The RMet package [42] uses a different processes such as segmentation to reduce unwanted peaks and defines the total number of metabolites. Importantly, RMet and TargetSearch give as output a list of total metabolites for a single sample, while gcProfileMakeR creates profiles based on large sets of samples (see results).

Furthermore, as sample analysis is very fast, datasets can be reanalysed helping in our understanding of metabolic regulation. Indeed, we have analysed our dataset at two different thresholds, one at 100% to define the constitutive set of volatiles (Figure 3 and Figure 4). As *co* does not produce a true constitutive profile, we compromised for a second threshold at 70% in order to obtain a set of volatiles for machine learning analysis (Table 1 and Table 2). This type of data exploration allows the through characterization of datasets that otherwise would be very difficult to implement by visual and manual analysis.

There are several reasons why data exploration using different thresholds for presence or absence is important. If a population shows the appearance of a certain compounds in some but not all the samples, it could point to differing sampling times, i.e., circadian effects, environmental effects such as thermo or photoperiod [38], or a genetic segregation of genes involved in the synthesis of a given compound [43]. By combining a quantitative threshold with genetic tools, this kind of scenarios can be sorted out from one another.

The data analysed show important insight in the regulation of scent emission. The floral scent profile of many plants can be used for phylogenetic analysis due to the robustness it shows [11,13]. We have defined the profile as the VOCs present in 100% of the samples of a given genotype, time of the day or treatment. It is remarkable that wild type flowers emit 13 volatiles while *co* emits 44, *def^nic^* 23 and *RNAi:AmLHY* 33. This suggests that the combination of the floral organ identity genes and clock genes create a shortlist of VOCs that shape the final composition of a given aroma. Importantly the number of constitutive VOCs i.e., profile is substantially smaller. This has significant implications for pollinator attraction and pest deterrence. Indeed, single volatiles such as limonene, myrcene and ocimene in *Mimulus* or benzaldehyde in *Capsella* play a key role in pollinator attraction [44,45]. The phenotypic gradient of floral identity goes from a wild type through a weak effect of *co* to a middle strong effect of *def^nic^* [5,46]. Indeed the PCA analysis shows a correlation with the aforementioned gradient, and places the *RNAi:AmLHY* lines closer to the wild type in terms of scent profile.

The analysis of gcProfileMakeR outputs by means of machine learning shows that SVM linear and RF algorithms identify the different genotypes with increased accuracy when the threshold is slightly relaxed i.e., 70% for constitutive profile. This is expected as the number of VOCS increases from 9 to 16, thus increasing the complexity of the samples. It is interesting to notice that the VOCs identified by RF as accurate for classification include acetophenone, which is a strong insect deterrent affecting pollination [47], and nonanal involved in attraction of beneficial insects [48].

The association of common and divergent VOCs between genotypes also opens the possibility of identifying cis-regulatory elements in key enzymes involved in single VOC synthesis with contrasting emission. This may help establish the molecular network coordinating metabolomes.

Finally, the analysis of complete metabolomes with tractable quality criteria is an important aspect of data reusability. Although a certain compound may not be found in all samples, it might be found in a subset. Thus, analysis of complete datasets that include the percentage of individuals of a given population producing a compound becomes possible. In this respect we think that using CAS numbers is an important asset as they are amenable to automatic analysis [29].

## 4. Materials and Methods

### 4.1. Plant Material

The *Antirrhinum majus* plants lines were grown in our lab since 2000. The *compacta (co)* mutant was obtained from IPK Gatersleben, while *deficiens-nicotianoides* (*def-nic*) was obtained from Zsuzsanna Schwarz-Sommer [49]. We used flowers from *Antirrhinum majus* wild type plants, *compacta* and *deficiens-nicotianoides* (*def-nic*) mutants [5] and *RNAi:AmLHY* from three independent transgenic lines described previously [8]. *Antirrhinum* plants were grown in the greenhouse using standard methods. Sampling periods of VOCs were 24 h for *def-nic* and *co*, while Sippe50 Wild type and *RNAi:AmLHY* were sampled every six hours for a complete day. The *RNAi:AmLHY* lines were aggregated to compare to other genotypes. We analysed 16 biological replicas for wild type Sippe 50, 35 for *co*, 9 for *def-nic* and 40 for *RNAi:AmLHY*.

### 4.2. GC-MS Analysis of Scent Profiles

Scent samples were analyzed in the following manner: we used flowers from *Antirrhinum majus* wild type (WT), the *compacta* (*co*) and *deficiens-nicotianoides* (*def-nic*) and *RNAi:AmLHY* from three independent lines. We collected volatiles from *co* and *def-nic* mutants for 24 h and a time course analysis was performed in WT and *RNAi:AmLHY* plants, where volatiles were sampled every 6 h for a complete day. In total, our experiment included 100 samples comprising 16 samples of WT, 35 of *co* mutant, 9 of *def-nic* mutant and 40 of *RNAi:AmLHY*. Fully developed flowers were introduced into desiccators containing 5% glucose in water to preserve humidity. The eMITTted VOCs were trapped in the headspace using clean Twisters^®^ (Gerstel, Mülheim an der Ruhr, Germany) covered with polyvinyl siloxane. Compounds adsorbed by the Twisters were analysed by a model HP-6890N GC–MS coupled to a 5975 mass spectrometer (Agilent Technologies, Palo Alto, CA, USA) combined with a TDU and cooling injector system (CIS4) (Gerstel).

Desorption of the Twisters was performed by heating from an initial temperature of 40° and increasing to 250 °C at 100 °C min^−1^ with 5 min hold time on splitless mode. Desorbed compounds were captured in a cool trap at − 100 °C. This process was automated by using an MPS2XL multipurpose sampler (Gerstel).

Chromatographic separation was done in a HP5MS-UI column (Agilent Technologies) with helium as gas carrier in constant pressure mode and split ratio 1:50. Initial temperature was 50 °C, increasing at a ratio of 5 °C min^−1^ until 70 °C held 1 min. In the next step, temperature was increased until 240 °C at 10 °C min^−1^ held for 15 min.

The mass spectrometer operated at 70 eV ionization voltage. Source and quadrupole temperatures were 230 and 150 °C, respectively. Mass range was 30.0 to 450.0 uma at 4 scan/s. MSD transfer line was maintained at 280 °C.

We used the ChemStation software (version E.02.02 SP1, Agilent Technologies) to acquire chromatograms. Compounds were qualitatively identified by comparison with the Wiley10th-NIST11b mass spectral database (Agilent Technologies, Wilmington, DE, USA).

### 4.3. Data Analysis

As our data set comprised four snapdragon groups (wild type, *RNAi:AmLHY* and the mutants *compacta* and *deficiens*), we divided the chromatograms in four folders for analysis with gcProfileMakeR. We passed the arguments to NormalizeWithinFiles function as follows. cas2rm takes a vector with CAS numbers from those compounds that should be removed, such as siloxane or related derivatives. We set the minimum quality to 80% (minQuality = 80). The getGroups function includes the pFreqCutoff parameter that ranges from 0 to 1.0 (default: 0.8) and we set it to 1.0 defining the “constitutive scent profile”. In this case, we focused on those compounds emitted by all analysed samples of a given group. Additionally, we defined another profile by setting pFreqCutoff to 0.7, which included those volatiles detected in more than 70% of the samples. For each defined profile and as we collected samples from four snapdragon groups, we obtained four scent profiles.

### 4.4. GC-MS Analysis of Scent Profiles

We performed a principal component analysis (PCA) [40]. Briefly, and as described previously [40], we calculated the relative amount of every volatile by dividing its area by total area of VOCs. Following the R code provided [40], we used a log ratio transformation before performing PCA.

We used and compared machine learning algorithms to classify scent profiles. This analysis was performed with the caret R package [50]. We used Naïve Bayes Classifier (NBC), k-Nearest Neighbor (k-NN), Support Vector Machine (SVM) and Random Forest (RF) [51,52,53,54]. The SVM algorithm have different methods, including linear and non-linear boundaries, such as kernel transformations [55,56]. We selected the following methods: SVM linear and the non-linear SVM radial and SVM polynomial, which allows to choose the optimal model across its parameters. As mentioned previously, to illustrate the gcProfileMakeR uS.A.-G.e we set the parameter pFreqCutoff to 1.0 and to 0.7. The data sets were randomly split into train (80%) and test (20%) sets using the createDataPartition function of the caret package. Then, each model was trained on the training set using 10-fold cross-validation. We compared the models with resamples function and we used accuracy and Kappa as metrics for multi-class classification, which are provided by caret package. Accuracy can be defined as the fraction of well-predicted samples over the total and Kappa, as Pa-Pe/1-Pe, where Pa is the fraction of well-predicted cases (as accuracy) and Pe is the concordance between observed and expected cases as if happening by chance.

We represented those volatiles that showed a quality above 80%, 59 VOCs in total, in a Venn diagram. The Venn diagram was plotted with the R/Bioconductor library “limma” [57] (R version: 3.6.1, package version: 3.42.2).

## Figures and Tables

**Table 1:** Comparative of machine learning algorithms. We used the metrics accuracy and Kappa, for comparing different algorithms in group 1 or constitutive scent profile (pFreqCutoff = 1.0) and group 2 (pFreqCutoff = 0.7). k-NN: k-Nearest Neighbors, NBC: Naïve Bayes Classifier, SVM: Support Vector Machine, RF: Random Forest, SD: standard deviation.

Metric	Algorithm	Group 1		Group 2	
		Mean	SD	Mean	SD
Accuracy	k-NN	0.77	0.17	0.9	0.14
	NBC	0.665	0.30	0.89	0.14
	SVM Linear	0.79	0.14	0.92	0.14
	SVM Radial	0.755	0.19	0.88	0.11
	SVM Polynomial	0.86	0.17	0.92	0.14
	RF	0.84	0.18	0.98	0.06
Kappa	k-NN	0.47	0.38	0.78	0.34
	NBC	0.52	0.37	0.75	0.32
	SVM Linear	0.53	0.40	0.82	0.34
	SVM Radial	0.52	0.39	0.78	0.18
	SVM Polynomial	0.65	0.42	0.81	0.34
	RF	0.61	0.46	0.95	0.14

**Table 2:** Random forest confusion matrix. The training set number of samples of each snapdragon group is shown in parentheses (observed column). The number of misclassified samples of each group are in columns (predicted columns). The class.error column indicates the percentage of misclassified samples (1-[(total correct predictions/total predictions) × 100]).

Group and pFreqCutoff	Observed	Predicted				Class.Error
		*compacta*	*deficiens*	*RNAi:AmLHY*	Wild type	
Group 1 (1.0)	*co* (28)	27	1	0	0	0.03
	*defnic* (8)	3	5	0	0	0.38
	*RNAi:AmLHY* (8)	1	0	7	0	0.13
	Wild type (4)	0	0	2	2	0.50
Group 2 (0.7)	*co* (28)	28	0	0	0	0
	*defnic* (9)	0	8	0	0	0
	*RNAi:AmLHY* (8)	1	0	6	1	0.25
	Wild type (4)	0	0	0	4	0

**Table 3:** Importance ranking of volatile organic compounds among Antirrhinum majus groups (wild-type, compacta mutant, deficiens mutant and RNAi:AmLHY) using random forest algorithm. The NIST library identifies two pairs of similar compounds which share the same retention time, 2′-Hydroxyacetophenone and 4′-Hydroxyacetophenone, and 2′-Methoxyacetophenone and 4′-Methoxyacetophenone, respectively. These compounds are depicted with a slash (“/”) in the table. Volatiles are ranked based on mean decrease in accuracy (MDA). This value indicates the accuracy in which a volatile can be used for classification.

Group 1 VOC	MDA	Group 2 VOC	MDA
Acetophenone	16.74	Nonanal	14.18
3,5-Dimethoxytoluene	16.09	Methyl-2-methylbutyrate	11.56
Methyl benzoate	12.53	Farnesene	11.49
Nonanal	11.73	Methyl benzoate	11.33
Ocimene	10.31	3,5-Dimethoxytoluene	11.18
Decanal	3.84	Acetophenone	10.59
Methyl-2-methylbutyrate	3.19	Phenethyl acetate	8.96
		Ocimene	8.80
		Methyl 3,5-dimethoxybenzoate	8.01
		Decanal	6.82
		Linalool	5.84
		2′-/4′-Hydroxyacetophenone	5.57
		Terpinolene	5.25
		Benzyl acetate	2.24
		Ethyl benzoate	0
		Nonanal	14.18

## Data Availability

The output data from Agilent Chemstation used as input and used for this study and output from gcProfileMakeR can be found as Supplementary Material (Chromatographic dataset). There are three additional dataset files in the application.

## Supplementary Materials

The following are available online at https://www.mdpi.com/article/10.3390/metabo11040211/s1: Figure S1: Non-constitutive scent profile by quality of wild-type, the mutants *compacta* and *deficiens-nicotianoides*, and the transgenic lines *RNAi:AmLHY*, Figure S2: Constitutive scent profile of wild-type, *compacta* and *deficiens* mutants and transgenic plants (*RNAi:AmLHY*) (pFreqCutoff = 0.7), Figure S3: Non-constitutive scent profile of wild-type, *compacta* and *deficiens* mutants and transgenic plants (*RNAi:AmLHY*), Figure S4: Chromatograms of wild type (a), *compacta* (b) and *deficiens* (c) mutants and *RNAi:AmLHY* (d), Figure S5: Non-constitutive scent profile by frequency of wild-type and transgenic *RNAi:AmLHY* snapdragons at four different time-points, denoted as ZT (zeitgeber time) 3, 9, 15 and 21, Table S1: List of removed siloxanes and their abstracts service registry numbers (CAS). Chromatographic dataset.
