# Supplementary material for: gcProfileMakeR: An R Package for Automatic Classification of Constitutive and Non-Constitutive Metabolites
Source: Metabolites. 2021 Mar 31;11(4):211. doi: 10.3390/metabo11040211 (PMC8065537; doi:10.3390/metabo11040211)
Supplement: Supplementary file 1 [file metabolites-11-00211-s001.zip › Supplementary_materials_data_Sanz_et_al/Perez-Sanz_Supplementary_Metabolites.docx]

**Supplementary Material**

gcProfileMakeR: an R package for automatic classification of constitutive and non-constitutive metabolites

Fernando Perez-Sanz ^1^, Victoria Ruiz-Hernández^2^, Marta I. Terry^3^, Sara Arce-Gallego^4^, Julia Weiss^3^, Pedro J Navarro^5^ and Marcos Egea-Cortines ^3^


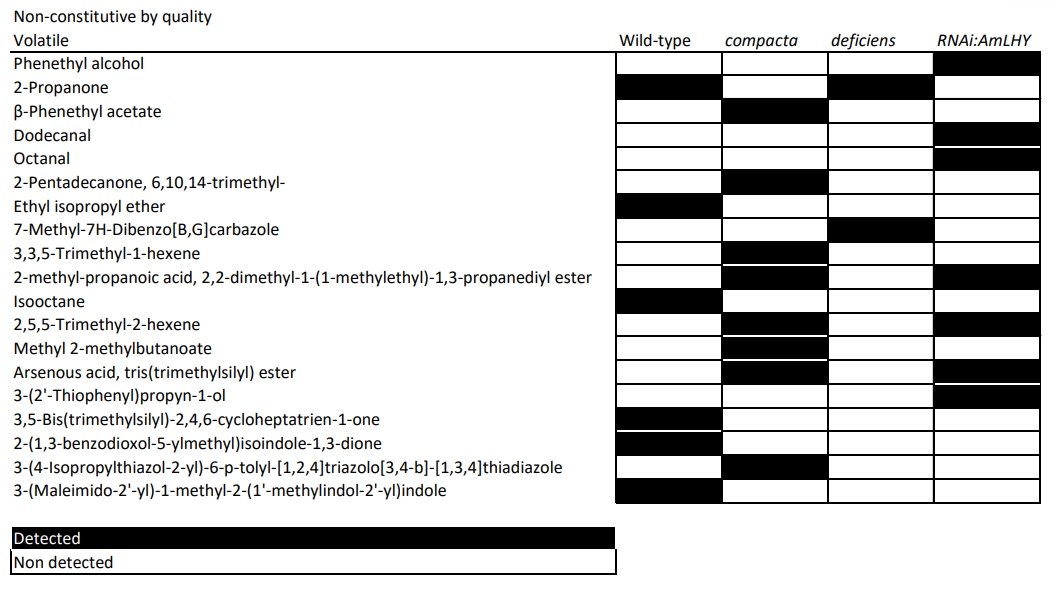


Figure S1: Non-constitutive scent profile by quality of wild type, the mutants *compacta* and *deficiens-nicotianoides*, and the transgenic lines *RNAi:AmLHY*. This profile comprises those compounds that had a quality lower than 80% (minQuality = 80, NormalizeWithinFiles function). Volatiles are listed according to their class. Black indicates detected compounds and white, non-detected compounds.


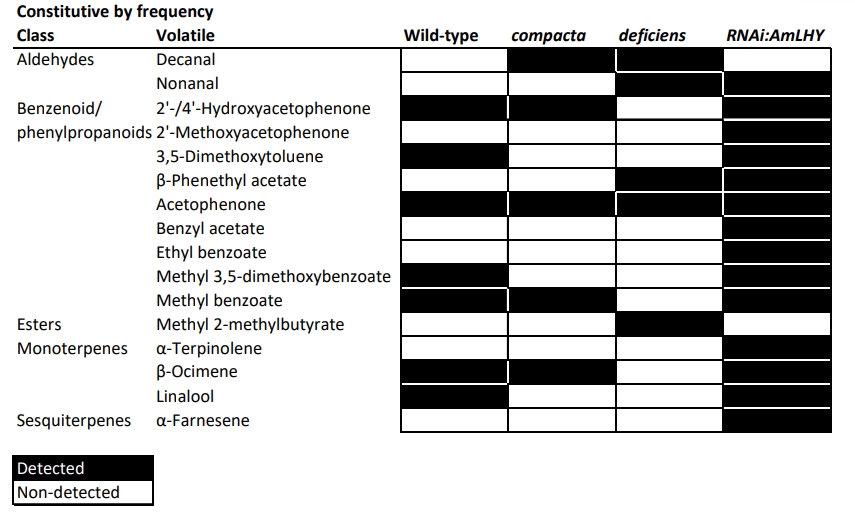


Figure S2: Constitutive scent profile of wild type, *compacta* and *deficiens-nicotianoides* mutants and transgenic plants (*RNAi:AmLHY*). Constitutive profile comprises those compounds that were present in 70% of analyzed samples. We set minQuality to 80% (NormalizeWithinFiles function). Volatiles are listed according to their class. Black cells indicate detected compounds and white, non-detected compounds.


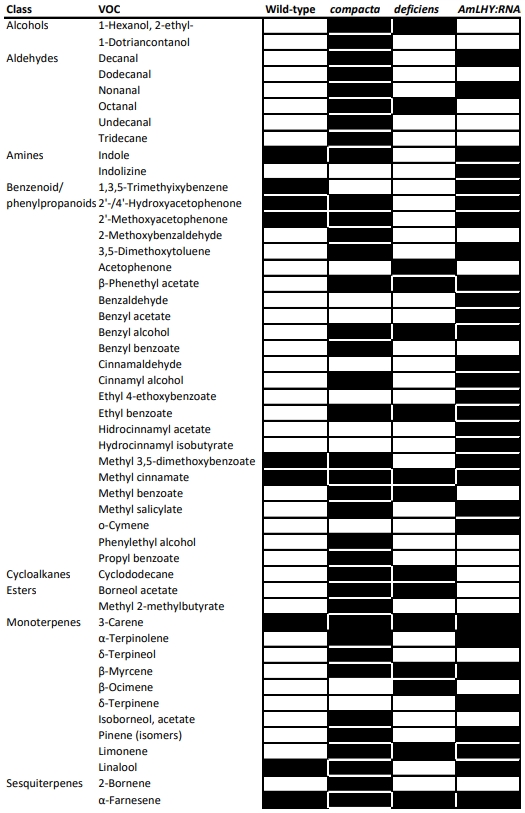


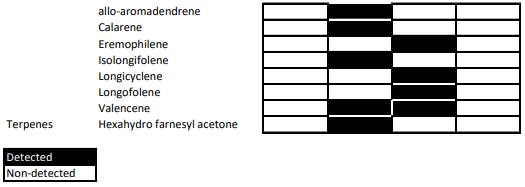


Figure S3: Non-constitutive scent profile of wild type, *compacta* and *deficiens-nicotianoides* mutants and transgenic plants (*RNAi:AmLHY*). This profile comprises volatiles that were present in less than 70% of samples. We set minQuality to 80% (NormalizeWithinFiles function). Volatiles are listed according to their class. Black indicates detected compounds and white, non-detected compounds.


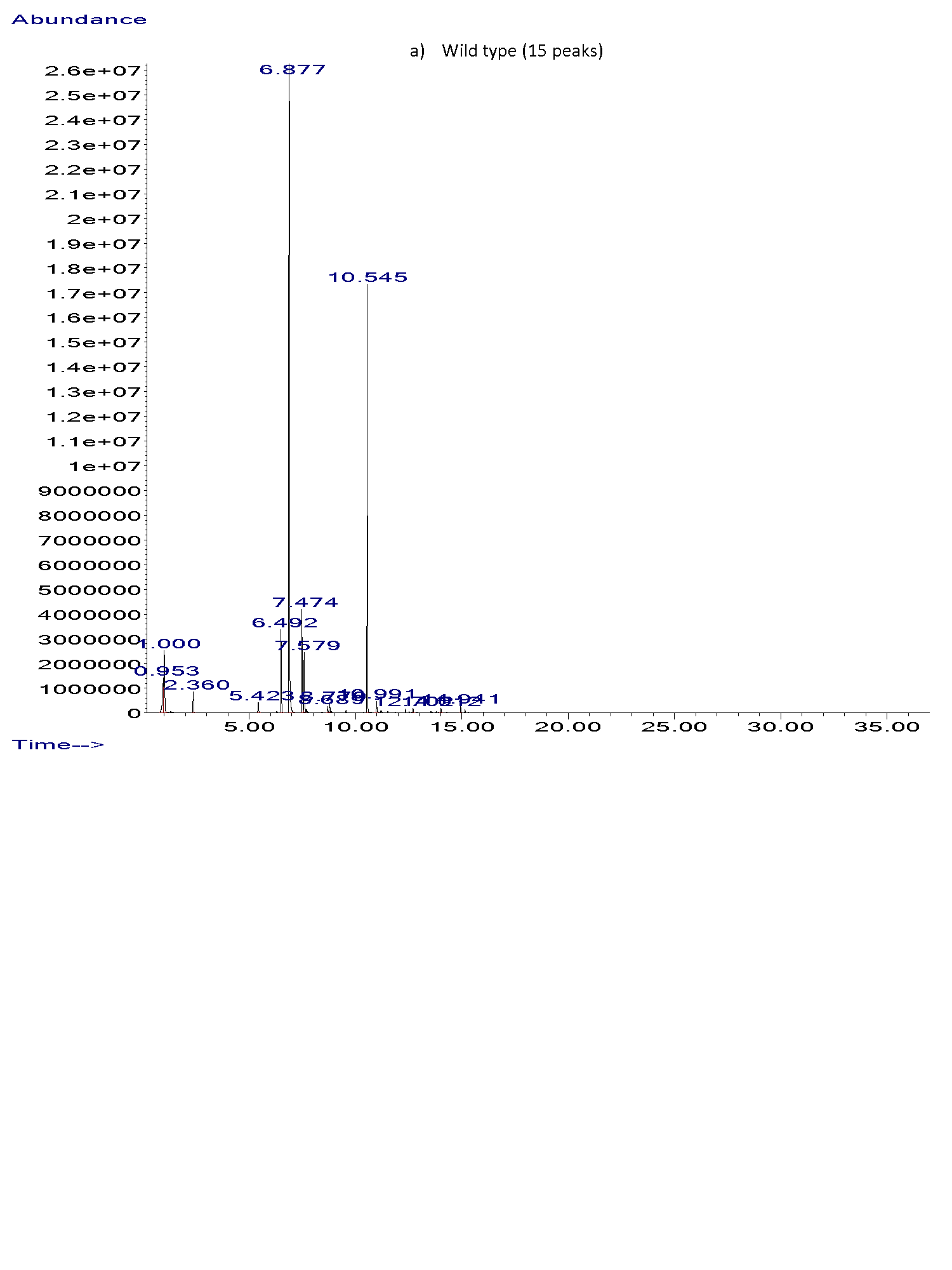


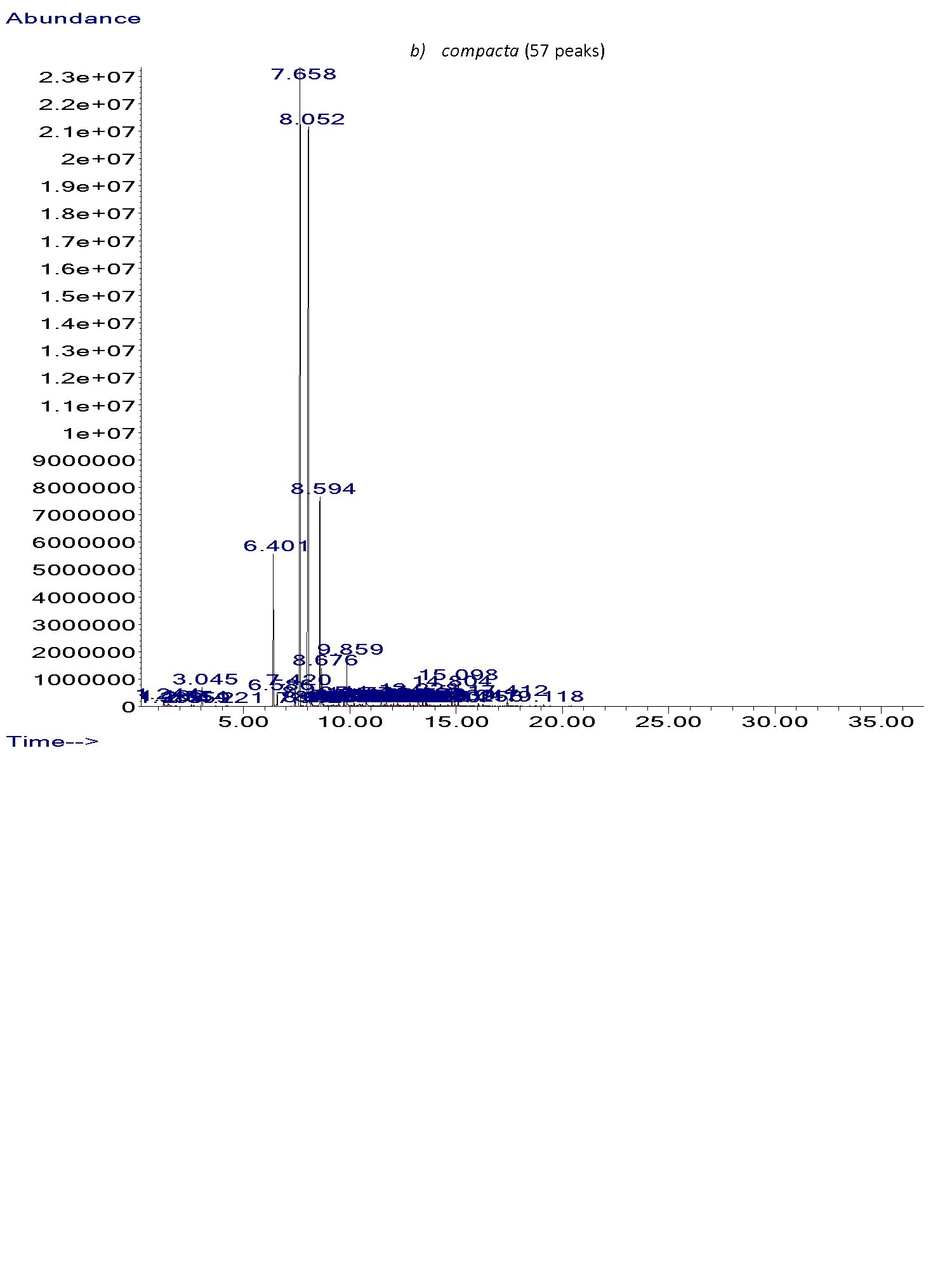


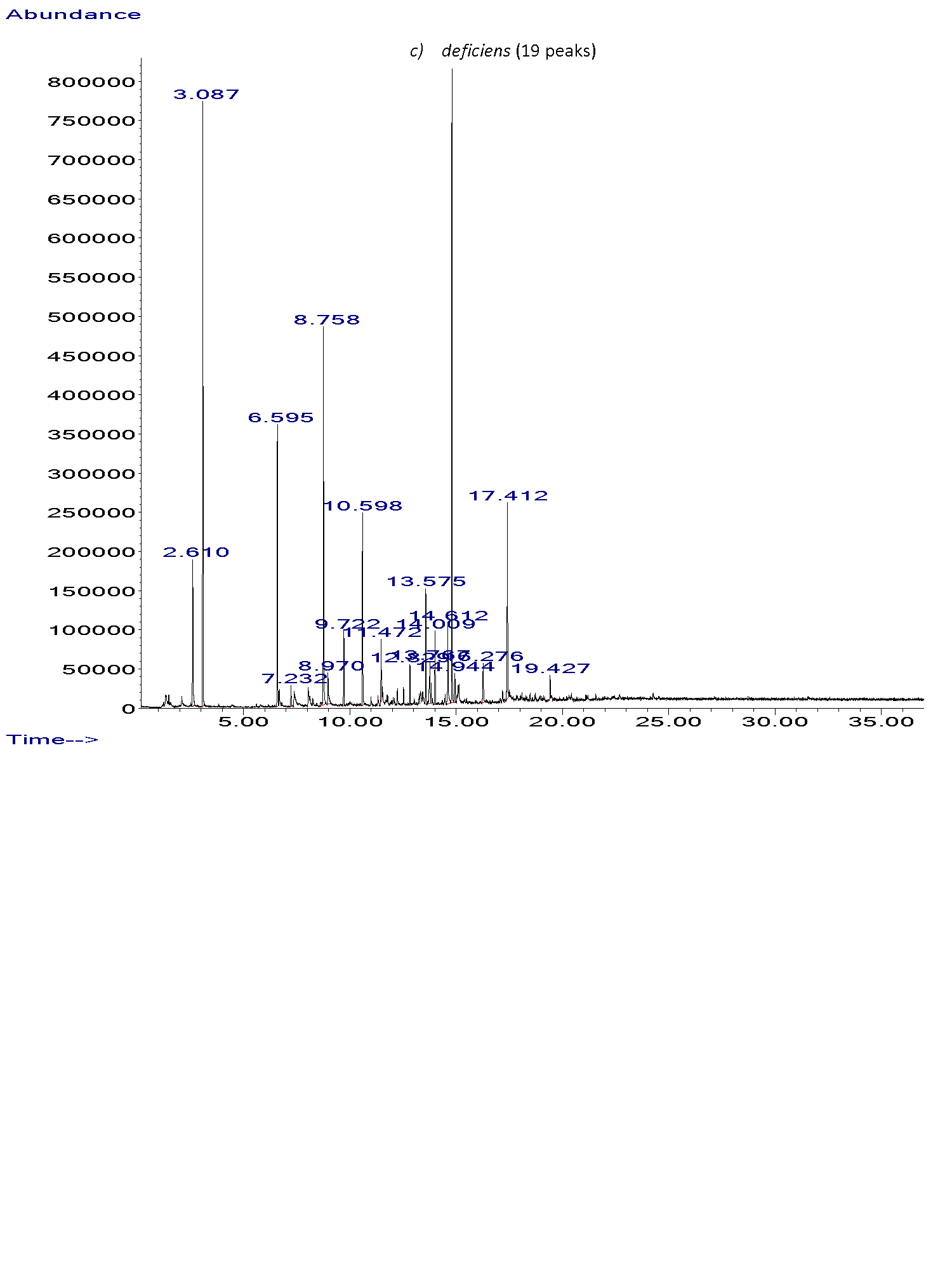


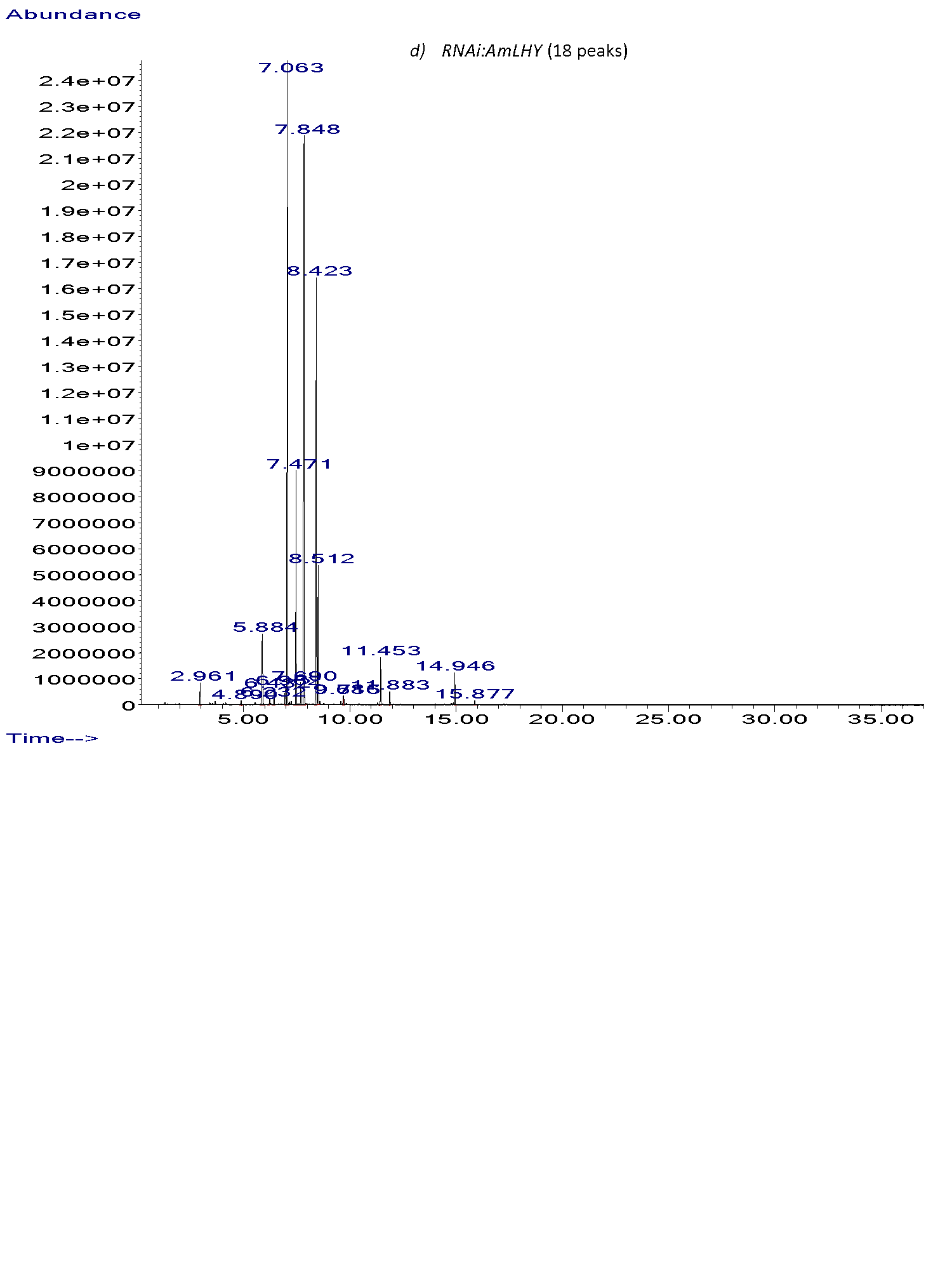


Figure S4: Chromatograms of wild type (a), *compacta* (b) and *deficiens* (c) mutants and *RNAi:AmLHY*


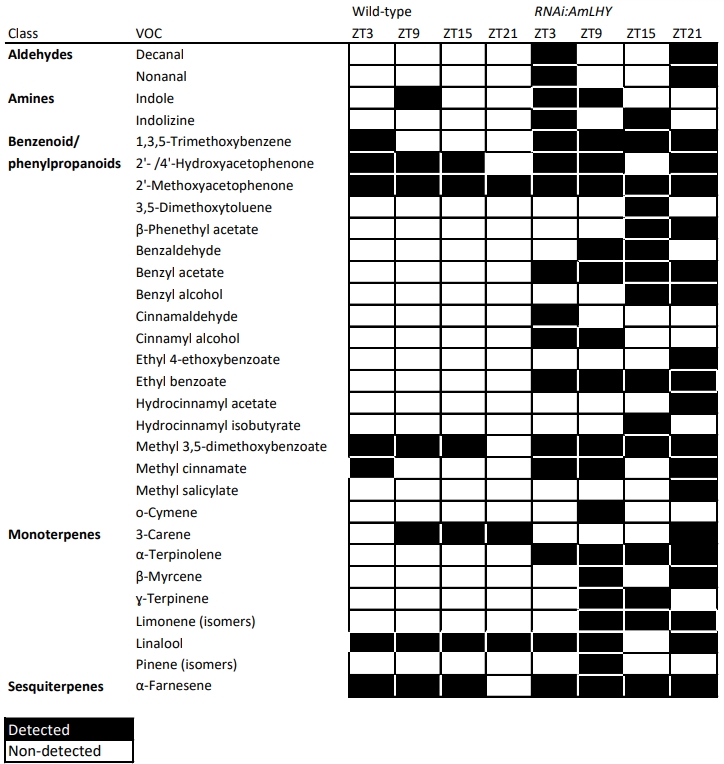


Figure S5: Non-constitutive scent profile by frequency of wild type and transgenic RNAi:AmLHY snapdragons at four different time points, denoted as ZT (zeitgeber time) 3, 9, 15 and 21. ZT0 represents the time of light son and ZT12, lights off. We set minQuality to 80% (NormalizeWithinFiles function). Constitutive profile includes VOCs that were present in 100% of analysed samples. Volatiles are listed according to their class. Black indicates detected compounds and white, non-detected compounds.

Table S1. List of removed siloxanes and their abstracts service registry numbers (CAS). The user can select which compounds should be removed by the filter “cas2rm” (NormaliceWithinFiles function). “cas2rm” uses as input data a vector that contains CAS numbers.

| CAS | Compound |
| --- | --- |
| 000540-97-6 | Dodecamethylcyclohexasiloxane |
| 000541-02-6 | Decamethylcyclopentasiloxane |
| 000541-05-9 | Hexamethylcyclotrisiloxane |
| 000556-67-2 | Octamethylcyclotetrasiloxane |
